# Modelling Remote Sensing Reflectance to Detect Dispersed Oil at Sea

**DOI:** 10.3390/s20030863

**Published:** 2020-02-06

**Authors:** Emilia Baszanowska, Zbigniew Otremba, Jacek Piskozub

**Affiliations:** 1 Department of Physics, Gdynia Maritime University, 81-225 Gdynia, Poland; 2 Institute of Oceanology, Polish Academy of Sciences, Powstańców Warszawy, 81-712 Sopot, Poland; piskozub@iopan.gda.pl

**Keywords:** dispersed oil, seawater, oil detection, remote sensing reflectance, spectral index

## Abstract

This paper presents a model of upwelling radiation above the seawater surface in the event of a threat of dispersed oil. The Monte Carlo method was used to simulate a large number of solar photons in the water, eventually obtaining values of remote sensing reflectance (R_rs_). Analyses were performed for the optical properties of seawater characteristic for the Gulf of Gdańsk (southern Baltic Sea). The case of seawater contaminated by dispersed oil at a concentration of 10 ppm was also discussed for different wind speeds. Two types of oils with extremely different optical properties (refraction and absorption coefficients) were taken into account for consideration. The optical properties (absorption and scattering coefficients and angular light scattering distribution) of the oil-in-water dispersion system were determined using the Mie theory. The spectral index for oil detection in seawater for different wind conditions was determined based on the results obtained for reflectance at selected wavelengths in the range 412–676 nm. The determined spectral index for seawater free of oil achieves higher values for seawater contaminated by oil. The analysis of the values of the spectral indices calculated for 28 combinations of wavelengths was used to identify the most universal spectral index of R_rs_ for 555 nm/440 nm for dispersed oil detection using any optical parameters.

## 1. Introduction

Detection and assessment of the amount of oil substances covering the sea surface is possible using various remote sensing methods in the wavelength range of ultraviolet, through the visible spectrum, infrared and microwaves up to radar waves. This issue has been described in several papers [1,2,3] and in great detail in papers and the monograph by Fingas [4,5,6]. On the other hand, substances under the surface of the water can only affect the above-water electromagnetic field in the visible range. Therefore, remote sounding of the sea column can only take place in the visible range. In large scale ocean optical measurements, the ocean colour is the key in practice. The colour of the seawater is caused by the interactions of incident solar light with substances or particles present in the water. Therefore, if oil is present in the water column, not only can the intensity of water-leaving light be changed, but the seawater colour may also differ. 

Taking into account natural environmental conditions, such as sea surface state, precipitation or sunshine, there is the possibility of natural or chemically-forced dispersion of a surface oil spill resulting in oil entering the seawater column. In addition, in the event of oil leaking from underwater mining and transfer installations, it is possible to disperse the oil already under the water surface. For example, this was done in 2010 when reducing the effects of a spill in the Gulf of Mexico [7]. Detection of oil dispersed in the water column is possible by means of an immersion oil sensor, for example, recording substances washed out of oil [8,9,10] or by means of a sensor analyzing light coming out of the sea. In the second case, the phenomenon of the interference of dispersed oil on the process of light transfer in the water column takes place [11,12]. Specifically, it is a change in the colour of the sea, manifested for spectrally varying changes in remote sensing reflectance R_rs_. This value is determined by the quotient of the above-water upwelling radiation L_u_(λ) to the above-water downwelling vector irradiance E_d_(λ) (for the same wavelength). 

In oceanographic measurement practice, instruments integrating an irradiance meter and a radiance meter would be used. This device, therefore, would record R_rs_. Assuming that such a multispectral device could be part of the sensor system for detecting dispersed oil in the sea, numerical simulations of light transfer in water containing oil contamination were conducted.

The radiative transfer model was applied which is based on the Monte Carlo method of simulating a large number of solar photons penetrating the sea surface (some of which return to the atmosphere and are registered by virtual photon detector [13,14,15]). As the input data for the model, the spectral distribution of both absorption and scattering coefficients, as well as angular distribution of light scattered on water density fluctuations and particles suspended in the water were used. The optical properties of seawater characteristic of the Gulf of Gdańsk (southern Baltic Sea) were adopted. In the model, the oil suspension modified the optical properties of the seawater. To simulate light transfer in oil-contaminated water, the optical properties of oil droplets had to be included. In this study, two types of oil that are extremely optically different were used. The performed analysis of the light transfer simulations allowed spectral indices of a defined sea area to be determined given the series of solar light wavelengths. The obtained results allowed the optimal spectral index to be found, whose value indicates the presence of oil dispersed in the seawater column.

## 2. Materials and Method

**Figure 1:**
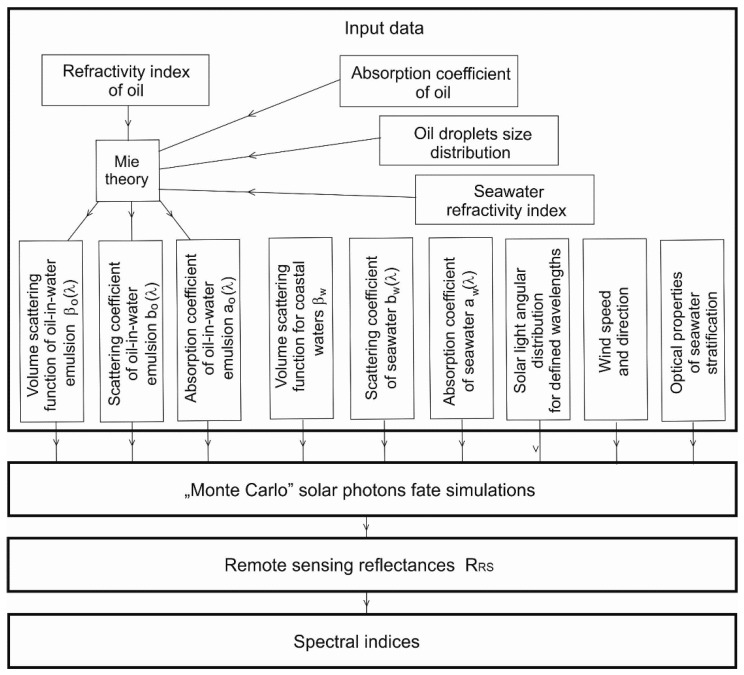
Block diagram of determining the spectral reflectance indices of the sea area unpolluted and with the addition of dispersed oil.

The process of searching for the appropriate spectral index for light reflectance starts with determining the optical properties of the seawater necessary for building the optical model of the sea area. The block diagram in Figure 1 shows the next stages of activities aimed at determining the spectral indices of reflectance of the unpolluted sea and the sea with the content of dispersed oil. The data regarding unpolluted seas were taken from the literature data (described in Section 2.2.2), while the data regarding the dispersed oil were obtained using the Mie solution of the light scattering on small particles (in this case, oil droplets).

The second stage—simulation of light energy transfer in seawater—is crucial for determining the differences between the spectral distributions of reflectance for a sea free of oil and a sea polluted with dispersed oils. In the third stage, combinations of reflectance quotients were compiled, taking into account all wavelengths for which the simulations were carried out. The fourth stage is a juxtaposition of the differences in spectral indices for a polluted sea with indices for an unpolluted sea.

### 2.1. Optical Model of the Sea Basin Studied

**Figure 2:**
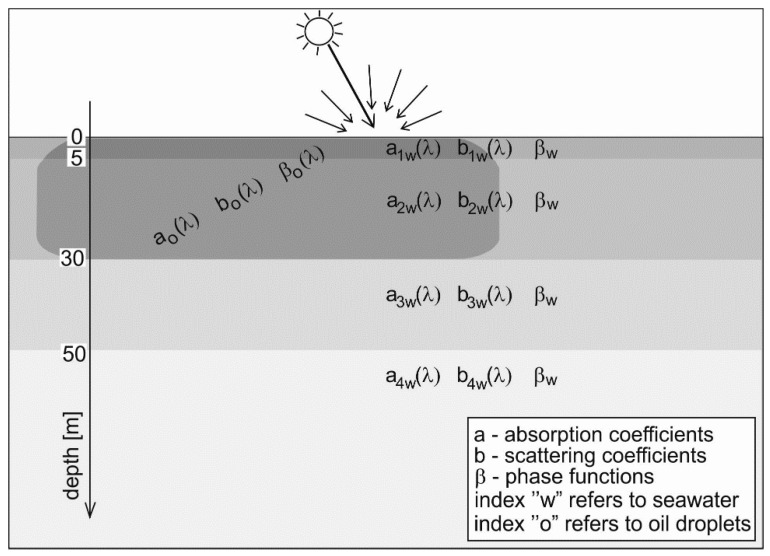
Optical model of the sea area.

The sea basin model was adopted based on the information available in the literature on the depth distribution of the absorption coefficient and the scattering coefficient typical for the Gulf of Gdańsk. Thus, a model of optical stratification of the sea is created as shown in Figure 2. The particular layers of seawater are described by the absorption coefficients a_1w_(λ), a_2w_(λ), a_3w_(λ), a_4w_(λ); scattering coefficients, b_1w_(λ), b_2w_(λ), b_3w_(λ), b_4w_(λ); and β_w_(λ), the scattering phase function. The oil dispersed in the seawater was in the sea layers from the sea surface to 30 m depth and is described by the absorption coefficients of oil a_o_(λ), scattering coefficients b_o_(λ) and the scattering phase function of oil β_o_(λ).

### 2.2. Input Data

#### 2.2.1. Solar Irradiance Distribution

The study included sunlight in a cloudless sky situation. Since the remote sensing reflectance may be significantly determined, not only by the optical properties of water but also by the angular distribution of (solar) light cast on sea surface, the share of diffused light in the atmosphere has been included in the downwelling light (Table 1). A sun height of 30 degrees was assumed.

#### 2.2.2. Optical Properties of the Seawater

The sea environment of the Baltic Sea depends on the human activities due to the sea being inland and shallow. Therefore optical properties of the Baltic Sea depends on the discharge of rivers and limited exchange with the waters of the North Sea.

The data on the optical properties of seawater: the absorption coefficient a_w_ and the scattering coefficient b_w_, as mentioned above, come from the work of Sagan [17], who presented results measured over several years in the southern Baltic Sea in the summer season (April–October), when an increase in phytoplankton concentration is observed. Table 2 contains the absorption coefficient data used in the simulation, while Table 3 includes the scattering coefficient. 

Angular scattering function after Petzold in the version for coastal waters was adopted in the model [18].

#### 2.2.3. Optical Properties of Oil-in-Water Emulsion 

The share of oil suspension in shaping the optical properties of water in the sea was determined based on the optical properties of oil and the typical size distribution of oil emulsion in water, which is schematically shown in Figure 1 as a block diagram. The properties of two extremely optically different oils were taken from the work of Otremba [19]. The oil droplet size distribution is determined experimentally in an oil-in-water emulsion prepared mechanically and stored for seven days. After this time, the emulsion changes its properties very slowly—the coagulation and coalescence processes almost disappear [20]. The inherent optical properties (IOPs) of the oil emulsion were determined using the Mie theory [21] for wavelengths for which the optical properties of seawater for the studied area are known. Input data for IOP calculations were taken from the literature: in the case when the optical properties of oils for different wavelengths were taken into account [19], and in the case when the oil droplets size distribution for the emulsion after 7 days of ageing was taken into account. The function describing size distribution as the exponential-logarithmic function (1) was used.

(1) $$\left(r\right)=A\exp\left[-\frac{\ln^{2}\left(r/r_{0}\right)}{2\sigma^{2}}\right]$$

where:

*A*—quantity parameter
*r*_0_—radius for maximum of distribution
*σ*—parameter of a shape of distribution

In this study *r*_0_ = 0.125 um, *σ* = 0.125 [20]. 

The optical properties of the oil suspension in seawater were determined (and later used in radiation transfer simulations) and are presented in Table 4 (absorption coefficient) and in Table 5 (scattering factor). The input data for the simulation includes VSF (volume scattering function) and tables of dispersed oil for the same wavelength as the absorption and scattering coefficients. 

## 3. Simulations

In radiance transfer simulations, the downwelling vector irradiance for a defined wavelength was represented by 200 million virtual photons. However, upwelling above-water radiance was represented by the number of photons falling into the conical solid angle 0.015697 sr sector divided by that solid angle. This solid angle corresponds to an 8.1-degree cone, which corresponds with the measuring angle for a cone of typical radiance meters. The simulations were conducted for a flat sea surface and for a rough sea caused by wind speeds of 2 m/s, 5 m/s and 10 m/s, in which the statistics of the slope angles are determined in accordance with the formulas given by Cox and Munk [22]. 

## 4. Results and Discussion

**Figure 3:**
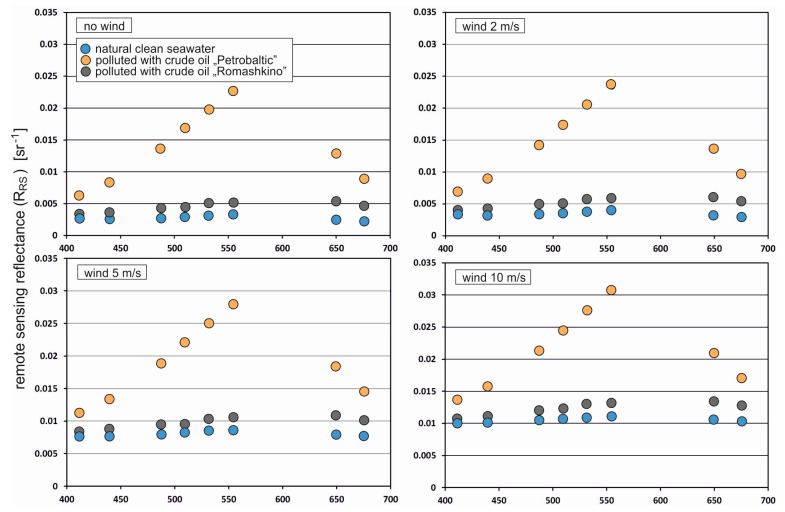
Remote sensing reflectance for natural seawater and seawater polluted by oil in concentration 10 ppm for Petrobaltic and Romashkino crude oils for calm sea surface and wavy surface as a result of wind with various speeds: 2 m/s, 5 m/s and 10 m/s, respectively.

The results of remote sensing reflectance R_rs_ for unpolluted seawater and the sea area polluted by dispersed Petrobaltic or Romashkino oils in a concentration of 10 ppm for no wind case and 2 m/s, 5 m/s and 10 m/s wind speeds, respectively, depending on light wavelengths are presented in Figure 3. The oil present in seawater causes an increase in remote sensing reflectance values for both considered crude oils. A significant increase of R_rs_ is determined for Petrobaltic crude oil and it is very likely that it is caused by low absorption coefficient values. For an above-water observer, this can be seen as an increase in the brightness of the sea surface. An analogous effect can also be caused by substances other than oil. Therefore, just increasing the brightness of the sea surface cannot be associated only with the presence of dispersed oil, because it can be caused, for example, by plankton bloom. A suspension of relatively transparent oil (in this case, Petrobaltic crude oil) increases the reflectance much more strongly than dark, opaque oil (in this case, Romashkino crude oil). The value of sea reflectance depends on all optical quantities that are input data for photon simulation and on the frequency of solar light reflections from wave slopes. An increase in reflectance is primarily caused by an increase in the scattering intensity and a decreased intensity of light absorption. Droplets of Petrobaltic crude oil explain a scattering coefficient greater for the whole visual spectrum than droplets of Romashkino crude oil (Table 4), while the opposite is true for the absorption coefficient (Table 3). 

The aim of the study was to identify a combination of wavelengths that result in oil detection, given optically extremely different oils. Therefore, based on the obtained results for R_rs_ at selected wavelengths for natural seawater and seawater polluted by dispersed oil, the spectral index was defined as the ratio of remote sensing reflectance for the longer wavelengths to the remote sensing reflectance for the lower wavelengths (formula (2)).

(2) $$I=\frac{R_{rs}(\lambda_{n})}{R_{rs}(\lambda_{m})}$$

where: *λ_n_*, *λ_m_* are higher wavelength and lower wavelength. 

**Figure 4:**
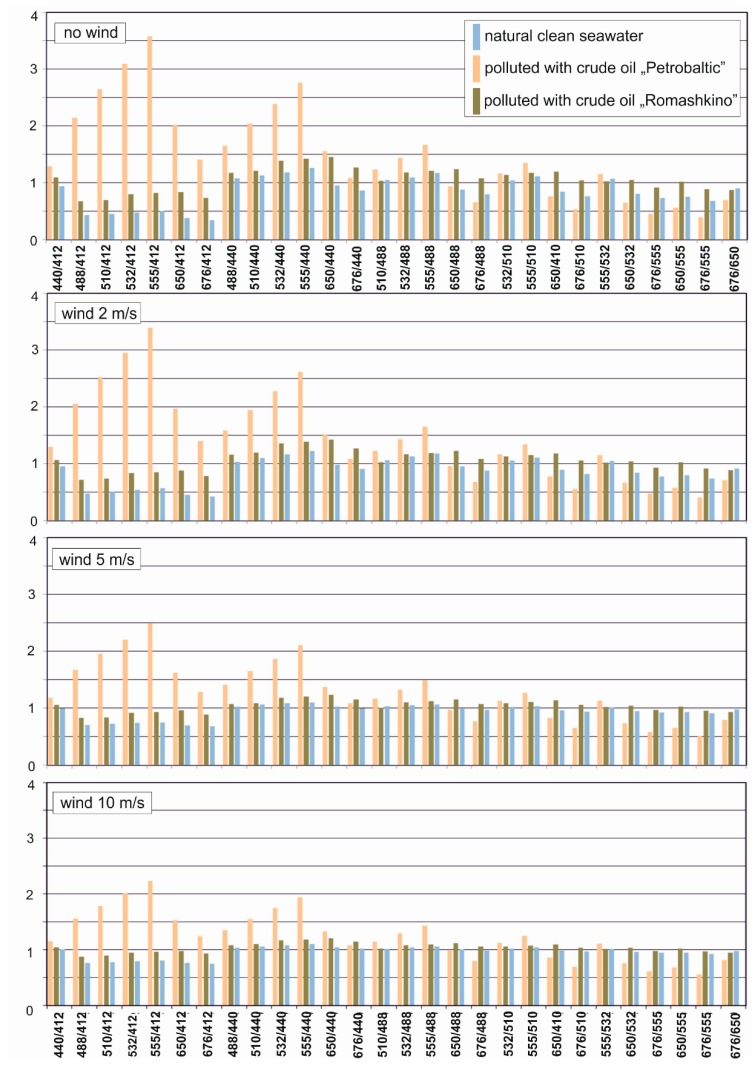
Spectral indices of the sea free of oil and in conditions of dispersed oil contamination for a calm sea surface and a wavy surface as a result of wind of various speeds (2 m/s, 5 m/s and 10 m/s, respectively).

**Figure 5:**
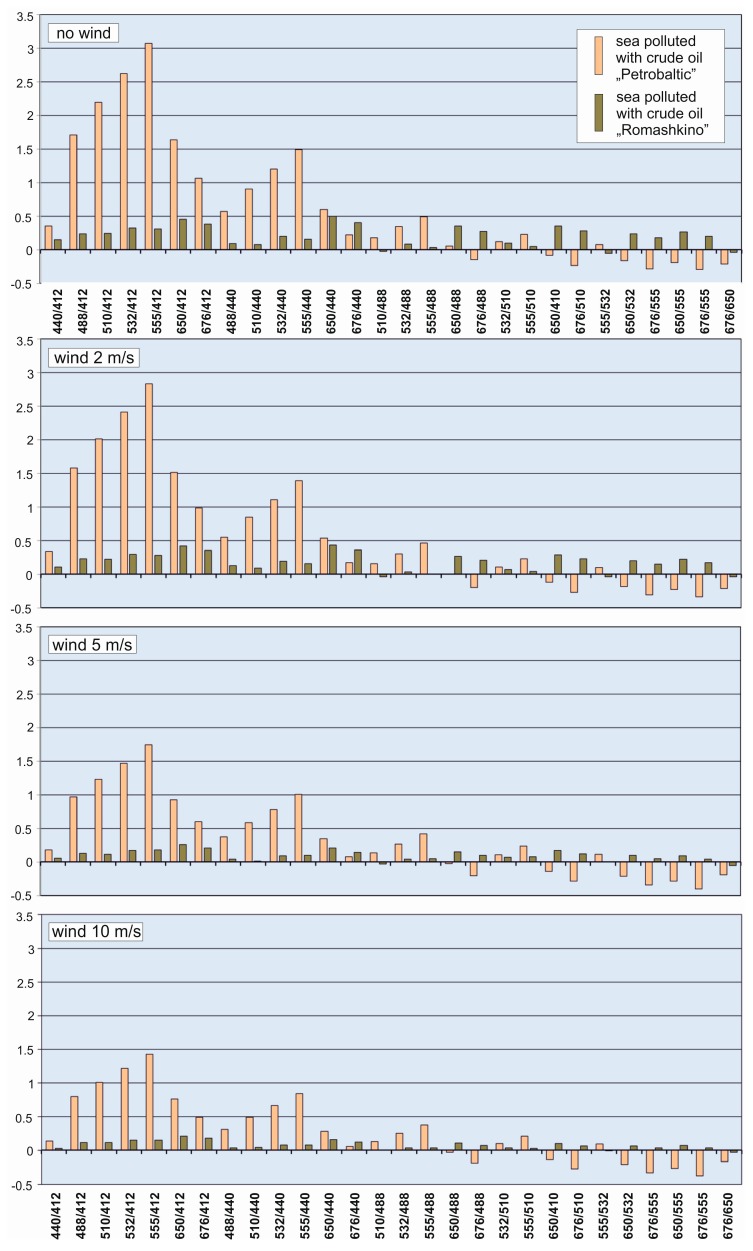
Differences between values of spectral indices of water contaminated by dispersed oil and values of indices of oil-free water contamination for a calm sea surface and a wavy surface as a result of wind of various speeds (2 m/s, 5 m/s and 10 m/s, respectively).

The spectral index was determined for natural seawater and seawater polluted by two types of oils for no wind case and 2 m/s, 5 m/s and 10 m/s wind speed, respectively, in the function of light wavelengths. Modelling of reflectance R_rs_ was carried out for 8 wavelengths, therefore 28 combinations of wavelengths for spectral index were performed. The results of all combinations of wavelengths for spectral index are shown in Figure 4. For Petrobaltic oil, it is a combination of 555/412 whereas for Romashkino oil, it was 650/440. However, the practical value of this information is limited, as reference should be made to the oil-free water reflectance. Therefore, for all combinations of wavelengths, oil index differences in relation to the unpolluted water index were determined, as shown in Figure 5. Taking into account the obtained results, 555/412 and 650/440 seem to be universal indices when both types of oil are considered simultaneously (Figure 5, upper chart). However, for a flat sea, the index 650/440 is more sensitive to the presence of Romashkino oil (upper chart), while for a wavy sea (bottom chart, wind 10 m/s) the index 650/412 is more sensitive. Since the complete absence of waving is unlikely in practice, an index of 650/412 would be recommended. Of course, if the sensor was dedicated to detecting only oil with optical properties similar to Petrobaltic oil, the index 555/412, 532/412 or 510/412 would be better. 

However, taking, as a rule, the universality of a sensor relative to different types of oil reflectance meter for wavelengths combination 412 nm and 650 nm, could be a part of the sensor system for detecting dispersed oil in the sea.

## 5. Conclusions

The studies described in this paper were aimed at determining the basic parameters of a possible above-water optical sensor designed to identify the presence of oil dispersed in the seawater column. Tracking a large number of virtual photons of sunlight falling on the model sea surface gives the opportunity to provide for the remote sensing reflectance (R_RS_) for a given region of the sea filled with either unpolluted or polluted water. The obtained results concern a sea basin (Gulf of Gdańsk), for which a two-channel remote sensing reflectance meter covering 555 nm and 440 nm would be suitable. On the other hand, if the criterion of similar values of the spectral index were adopted for various types of oil, then the 650/440 index would be optimal. However, in this case, the value of such index is relatively low. In other regions of the sea, if the optical properties of unpolluted water differ from those included in this paper—and if inherent optical properties (IOPs) for define sea region are available—the reflectance simulations can be performed in a similar way as in this study. It should also be noted that the results of the analyses shown in this paper relate to a cloudless sky. It is possible that on a cloudy day, another spectral index may prove to be effective. It is useful despite the surface of the sea being rough, which reduces the ability to detect the oil suspension, but does not make it impossible. 

## Figures and Tables

**Table 1:** Direct and diffuse solar irradiance contributions for various light wavelengths (RADTRAN model developed by Gregg and Calder [16]).

Wavelength [nm]	Diffuse Solar Irradiance Fraction	Direct Solar Irradiance Fraction
412	0.353	0.647
440	0.338	0.662
488	0.318	0.682
510	0.312	0.688
532	0.306	0.694
555	0.301	0,699
650	0.288	0.712
676	0.286	0.714

**Table 2:** Absorption coefficient a_w_ of seawater free of oil droplets for various light wavelengths at various sea depths [17].

λ (nm)	Absorption Coefficient [m^−1^]			
	Depth < 5 m	5–30 m	30–50 m	Depth > 50 m
412	1.096	0.656	0.536	0.546
440	0.788	0.428	0.338	0.338
488	0.448	0.228	0.168	0.178
510	0.358	0.188	0.148	0.148
532	0.293	0.163	0.133	0.143
555	0.249	0.149	0.129	0.129
650	0.441	0.391	0.371	0.371
676	0.637	0.507	0.477	0.477

**Table 3:** Scattering coefficient (b_w_) of seawater free of oil droplets for various light wavelengths at various sea depths [17].

λ (nm)	Depth < 5 m	5–30 m	30–50 m	Depth > 50 m
412	1.48	0.62	0.33	0.37
440	1.01	0.6	0.32	0.36
488	0.97	0.6	0.32	0.35
510	0.98	0.6	0.32	0.36
532	0.96	0.6	0.32	0.35
555	0.92	0.59	0.32	0.32
650	0.91	0.58	0.29	0.32
676	0.82	0.53	0.29	0.32

**Table 4:** Absorption coefficient a_o_ of oil droplets dispersed in water in a concentration of 10 ppm for various light wavelengths.

λ (nm)	Dispersed Oil Petrobaltic [m^−1^]	Dispersed Oil Romashkino [m^−1^]
412	0.299	2.3
440	0.114	1.96
488	0.052	1.49
510	0.042	1.33
532	0.029	1.14
555	0.029	1.04
650	0.0125	0.75
676	0.0087	0.71

**Table 5:** Scattering coefficient b_o_ of oil droplets dispersed in water in a concentration of 10 ppm for various light wavelengths.

λ (nm)	Dispersed Oil Petrobaltic	Dispersed Oil Romashkino
412	7.81	5.6
440	7.97	5.9
488	7.98	6.45
510	7.95	6.65
532	7.91	6.83
555	7.87	6.89
650	7.6	7.01
676	7.48	6.9

## References

[1] Hu C., Feng L., Holmes J., Swayze G.A., Leifer I., Melton C., Garcia O., Macdonald I., Hess M., Muller-Karger F. (2018). Remote sensing estimation of surface oil volume during the 2010 Deepwater Horizon oil blowout in the Gulf of Mexico: Scaling up AVIRIS observations with MODIS measurements. J. Appl. Remote Sens..

[2] Sun S., Hu C., Feng L., Swayze G.A., Holmes J., Graettinger G., MacDonald I., Garcia O., Leifer I. (2016). Oil slick morphology derived from AVRIS measurements of the Deepwater Horizon oil spill: Implications for spatial resolution requirements of remote sensors. Mar. Pollut. Bull..

[3] Hou Y., Li Y., Liu B., Liu Y., Wang T. (2017). Design and Implementation of a Coastal-Mounted Sensor for Oil Film Detection on Seawater. Sensors.

[4] Fingas M., Brown C. (2014). Review of oil spill remote sensing. Mar. Pollut. Bull..

[5] Fingas M., Brown C. (2018). A Review of Oil Spill Remote Sensing. Sensors.

[6] Fingas M. (2017). Oil Spill Science and Technology.

[7] Murawski S.A., Ainsworth C.H., Gilbert S., Hollander D.J., Paris C.B., Schlüter M., Wetzel D.L. (2019). Deep Oil Spills: Facts, Fate, and Effects.

[8] Baszanowska E., Otremba Z. (2017). Fluorometric index for sensing oil in the sea environment. Sensors.

[9] Baszanowska E., Otremba Z. (2019). Detecting the Presence of Different Types of Oil in Seawater Using a Fluorometric Index. Sensors.

[10] Baszanowska E., Otremba Z. (2015). Modification of optical properties of seawater exposed to oil contaminants based on excitation-emission spectra. J. Eur. Opt. Soc. Rap. Public..

[11] Otremba Z., Król T. (2002). Modelling of the crude oil suspension impact on inherent optical parameters of the coastal seawater. Pol. J. Environ. Stud..

[12] Haule K., Freda W., Darecki M., Toczek H. (2017). Possibilities of optical remote sensing of dispersed oil in coastal waters. Estuar. Coast. Shelf Sci..

[13] Piskozub J., Weeks A.R., Schwartz J.N., Robinson I.S. (2000). Self-shading of upwelling irradiance for an instrument with sensors on a sidearm. Appl. Opt..

[14] Otremba Z., Piskozub J. (2003). Modelling of the optical contrast of an oil film on a sea surface. Opt. Express.

[15] Rudz R.K., Darecki M., Toczek H. (2013). Modelling the influence of oil content on optical properties of seawater in the Baltic Sea. J. Eur. Opt. Soc. Rap. Public.

[16] Gregg W.W., Carder K.L. (1990). A simple spectral solar irradiance model for cloudless maritime atmospheres. Limnol. Oceanogr..

[17] Sagan S. (2008). The inherent water optical properties of Baltic waters. Rozprawy i Monografie.

[18] Petzold T.J., Tyler J.E. (1977). Volume scattering functions for selected ocean waters. Light in the Sea.

[19] Otremba Z. (2000). The impact on the reflectance in VIS of a type of crude oil film floating on the water surface. Opt. Express.

[20] Otremba Z. (2007). Oil droplets as light absorbents in seawater. Opt. Express.

[21] Bohren C.F., Huffman D.R. (1983). Absorption and Scattering of Light by Small Particles.

[22] Cox C., Munk W.H. (1954). Statistics of the sea surface derived from sun glitter. J. Mar. Res..

